# Molecular Determinants of Glioblastoma Response to Epidermal Growth Factor Receptor Kinase Inhibitors

**DOI:** 10.4137/cmo.s393

**Published:** 2008-03-19

**Authors:** Ricardo J. Komotar, Marc L. Otten, Maxwell B. Merkow, Jeffrey N. Bruce

**Affiliations:** Department of Neurosurgery, Columbia University, New York, NY

Despite the improved prognosis for many cancer patients, the survival for those with Glioblastoma multiforme (GBM) remains dismal. Even with aggressive intervention, including resection, radiation, and chemotherapy, the overall 2-year survival rate is only 25% in the most optimistic series, and 5-year survival rates are consistently in the low single digits (1, 2). Therefore, it is evident that novel therapeutic paradigms are necessary to overcome the inherent limitations of conventional treatments. Emerging data offer encouraging evidence that patient-specific therapies tailored to the unique biology of an individual’s GBM may improve clinical outcomes.

EGFR is a receptor tyrosine kinase (RTK) that is commonly overexpressed in GBMs, and this overexpression typically occurs as a result of gene amplification. The EGFR-mediated signal transduction results in activation of a number of downstream pathways including PI3K-AKT and RAS-MAPK, inhibiting apoptosis and driving proliferation. Several studies have demonstrated variability of gene expression in different sets of GBMs (3–5). In particular, tumors expressing EGFR, which is associated with a particularly poor prognosis, can clearly be identified by microarray expression analysis (5). Alternatively, deletion within the EGFR coding sequence can result in the expression of a truncated, mutant EGFR protein that signals constitutively. With the development of molecular inhibitors targeting multiple pathways, such as EGFR, the combination of expression analysis and mutation status may enable the development of therapeutic modalities tailored to individual disease characteristics.

To this end, Mellinghoff and colleagues investigated the molecular determinants of GBM responsiveness to EGFR kinase inhibitors (6). In this study, the authors sequenced the kinase domains of EGFR and human EGFR type 2 (Her2/neu). They also analyzed the expression of EGFR, EGFR deletion mutant variant III (EGFRvIII), and phosphatase and tensin homolog (PTEN) in recurrent malignant gliomas from 26 patients receiving EGFR kinase inhibitor (EGFR-ki) therapy. Twenty-seven percent of patients (7/26) responded to EGFR-ki therapy by showing tumor shrinkage of at least 25%. All others had rapid disease progression. There were no mutations in EGFR or Her2/neu detected. Coexpression of EGFRvIII and PTEN, however, was significantly associated with clinical responsiveness (P < 0.001; OR 51). These findings were validated in 33 GBM patients undergoing similar treatment at a different institution (P = 0.001, OR 40). Furthermore, in vitro coexpression of EGFRvIII and PTEN by GBM cells was associated with sensitivity to erlotinib. The authors concluded that the presence of EGFRvIII and PTEN may serve as predictive markers for treatment success in GBM patients receiving EGFR kinase inhibitors.

The authors have elucidated rational therapeutic targets, thereby enhancing clinical applicability and adding to an increasing body of literature supporting the utility of expression profiling in GBM patient stratification (7, 8). From their findings it is apparent that adjuvant regimens should not be uniform, and that choosing the most efficacious agent for each individual may require routine expression analysis to target susceptible molecular pathways. For instance, expression analysis could be conducted immediately after resection in multiple biopsy samples of core and invasive tumor regions, perhaps identified on preoperative magnetic resonance imaging (MRI). Implementing expression analysis as a standard clinical test, however, will require significant changes in the way clinicians approach GBM treatment, as well as the development of substantial infrastructure for rapid, high-throughput expression and mutation analysis. The subsequent challenge of interpreting this data will require an expansion of expertise from academic studies to wider clinical practice. Despite these obstacles, it is vital to take into account the genetic mutations present in each patient’s tumor before chemotherapeutic agents that target molecular pathways can ever be expected to achieve maximal clinical efficacy. In conclusion, it is important to consider GBMs as a group of related but genetically distinct tumor cells, and capitalize on this knowledge by using therapies that target all populations present within the lesion.
